# Preliminary Validation of the Italian Night Eating Questionnaire (I-NEQ-16): Item Analysis and Factor Structure

**DOI:** 10.3389/fpsyg.2018.02628

**Published:** 2018-12-20

**Authors:** Marco Innamorati, Claudio Imperatori, David Lester, Mariantonietta Fabbricatore, Lavinia Gaudini, Anna Contardi, Michela Balsamo

**Affiliations:** ^1^Department of Human Sciences, European University of Rome, Rome, Italy; ^2^Department of Psychology, Stockton University, Galloway, NJ, United States; ^3^Department of Psychological, Health, and Territorial Sciences, G. d’Annunzio University Chieti–Pescara, Chieti, Italy

**Keywords:** night eating syndrome, night eating questionnaire, night eating diagnostic questionnaire, structural equation modeling, ROC curves

## Abstract

Night eating syndrome (NES) severity is usually assessed with the Night Eating Questionnaire (NEQ). Although the most common version of the NEQ is composed of 14 items (NEQ-14), two additional items measuring distress associated with the night eating have been proposed, but they have never been included in past psychometric studies. The aim of the present study was to create an Italian version of the NEQ-16 (I-NEQ-16), with the inclusion of the proposed items for assessing the distress associated with night eating. A major objective of the study was to propose a unidimensional version of the I-NEQ-16 and investigate its psychometric properties. 482 Italian adults (380 women and 102 men; mean age = 25.5, *SD* = 10.9 years old) were administered the Italian versions of the NEQ, the Night Eating Diagnostic Questionnaire (NEDQ), and questionnaires measuring binge eating, emotional and external eating, diurnal chronotype, insomnia, and anxiety and depression severity. In order to improve the unidimensionality of the I-NEQ-16, we removed from further analyses items 1, 4, and 7, because they increased the heterogeneity of the measure. Confirmatory factor analysis, indicated the fit of a modified one-factor model, allowing correlated errors between three pairs of items. I-NEQ-16 scores were significantly associated with all concurrent questionnaire scores and were able to categorize individuals according to their diagnosis of NES according to the NEDQ. Thus, the I-NEQ-16 is a valid measure that is potentially useful for investigating correlates of night eating in the general population.

## Introduction

Night eating syndrome (NES) is included in the Otherwise Specified Feeding and Eating Disorders section of the Diagnostic and Statistical Manual of Mental Disorders–5 (DSM-5) (American Psychiatric Association, 2013) and is characterized by morning loss of appetite, evening hyperphagia, repeated binge eating episodes during the night, and insomnia (Stunkard et al., 1955; Birketvedt et al., 1999; Allison et al., 2010). The syndrome is characterized also by awareness and recall of the night eating and significant distress and/or impairment in the functioning of the individual (American Psychiatric Association, 2013). The prevalence of NES in the general population is around 1.1–1.5% (Rand et al., 1997; de Zwaan et al., 2014; Olejniczak et al., 2018), and could be higher (between 2.8 and 12.4%) in nonclinical populations of university students, with significant variation depending on the measures used to collect information (Runfola et al., 2014; Yahia et al., 2017; He et al., 2018). Wide variations in NES prevalence have also been reported in samples of overweight and obese patients (Aronoff et al., 2001; Allison et al., 2007; Calugi et al., 2009), with one study identifying 51% of patients with severe obesity also satisfying the criteria for NES (Aronoff et al., 2001) according to the modified criteria proposed by Stunkard et al. (1996) (i.e., morning anorexia, evening hyperphagia and insomnia).

NES scores are strongly associated with eating psychopathology (de Zwaan et al., 2014), especially binge eating disorder (Napolitano et al., 2001; Grilo and Masheb, 2004; Colles et al., 2007; Allison et al., 2008; McCuen-Wurst et al., 2018) and with psychiatric disorders (Civil Arslan et al., 2015; Saracli et al., 2015; Kim et al., 2016; Melo et al., 2018). For example, de Zwaan et al. (2006) reported that, of 106 individuals with nighttime eating problems (i.e., individuals who self-reported “eat large amounts of food in the evenings and/or get up during the night to eat”), around 28% were currently taking antidepressants and 55.7% had lifetime major depression (assessed through a semi-structured clinical interview). Assessing both the prevalence and severity of the NES is an important public health goal because it is frequently associated with metabolic disorders (e.g., type 2 diabetes; Allison et al., 2007), and worse outcomes for nutritional treatments in obese patients (Gluck et al., 2001).

Currently, NES severity is usually assessed with the Night Eating Questionnaire (NEQ) (Allison et al., 2008), the global score of which is used to screen for the possible presence of NES. Recently, another instrument, the Night Eating Diagnostic Questionnaire (NEDQ) (Gluck et al., 2001; Nolan and Geliebter, 2017), has been proposed as a diagnostic tool for NES.

The most common version of the NEQ is composed of 14 items (NEQ-14) measured on a five-point Likert-type scale, although item 13 is considered to be only a screening tool for differentiating the NES from the parasomnia sleep-related eating disorder, and it is commonly not considered when measuring NES severity or assessing the factorial structure of the scale. Two additional items measuring distress associated with the night eating (i.e., “How upsetting is your night eating to you?” “How much has your night eating affected your life?”) have been proposed by Allison et al. (2008), but they have not generally been included in the versions of the NEQ used in research. In a validation study, Allison et al. (2008) used an exploratory/confirmatory factorial approach to investigate the dimensionality of the NEQ-14. This resulted in a four-factor solution (Nocturnal Ingestions, Evening Hyperphagia, Morning Anorexia, and Mood and Sleep Disturbance). Subsequent confirmatory factor analyses were conducted to assess the appropriateness of a total score. The authors compared the fit of three factorial models: (1) a one-factor model; (2) a four-factor model; and (3) a hierarchical model with items loading on four first-order factors and one single higher-order factor. Only the second and the third models fitted the data well. Nevertheless, a ratio of the chi-square values (χ^2^; model 2/model 3) of 98.93 was considered suggestive of the presence of a higher-order factor and the appropriateness of the use of a global score. Cronbach alpha for the total scale was 0.70, while internal consistencies for the four factors ranged between 0.30 for Mood and Sleep Disturbance and 0.94 for Nocturnal Ingestions. However, it should be noted that three of the four factors had alphas <0.70. In a second study conducted with a sample of 81 patients with NES, NEQ-14 scores were significantly correlated with measures of insomnia, depression, and perceived stress (Allison et al., 2008).

Subsequent international studies (Moize et al., 2012; Elsadek et al., 2014; Latzer et al., 2014; Meule et al., 2014; Tu et al., 2017) investigating the factor structure of the NEQ-14 have consistently reported a multidimensional structure that is not always consistent with the factor structure reported by Allison et al. (2008). Moreover, these studies did not investigate the possible presence of a higher-order factor, used orthogonal rotation procedures which impose the restriction that the components be uncorrelated, and consistently used controversial factorial methods (i.e., they used a PCA which extracts components from common and unique variance which is not considered to be a factorial method). Although these studies proposed a multidimensional structure for the NEQ-14, they all proposed calculation and use of a global score. In Italy, a version of the NEQ-14 has been used in correlational studies (e.g., Dalle Grave et al., 2013; Vinai et al., 2015), and only recently have factor structure and psychometric properties been investigate (Aloi et al., 2017). Aloi et al. (2017) submitted an Italian version of the NEQ-14 to 574 adults and evaluated the fit of a hierarchical model consistent with the model tested by Allison et al. (2008). The authors reported the fit of this model, but low alphas for the first order factors (from 0.48 to 0.71) and the higher-order factor (0.65).

One limitation of the NEQ-14 is the fact that not all the proposed criteria for a diagnosis of NES are assessed with this version of the questionnaire (Runfola et al., 2014). For example, distress associated with night eating is assessed only by the two additional items proposed by Allison et al. (2008), and it is surprising that, as far as we know, past studies have not investigated the psychometric properties of the 16-item version of the NEQ (NEQ-16). Additional items proposed by Allison et al. (2008) for assessing distress or impairment have also rarely been used in past research. For example, Nolan and Geliebter (2017), who investigated the convergent validity for the NEDQ with the NEQ, rated NES severity with the NEQ-14 and evaluated the presence of distress or impairment as a result of night eating with items from the NEDQ and not with the items suggested by Allison et al. (2008).

Considering that distress associated with night eating is an important criterion when screening for the possible presence of NES, and the lack of validated Italian versions of the NEQ-16, the main aim of the present study was to create an Italian version of the NEQ-16 (I-NEQ-16), which includes the two items proposed by Allison et al. (2008) for assessing the distress associated with night eating. Furthermore, considering that unidimensionality, the basis for using a NEQ global score for rating NES severity, has always been considered critical in past research, another aim of the study was to propose a unidimensional version of the I-NEQ-16 and to investigate its psychometric properties. Unlike past research by others (e.g., Moize et al., 2012; Elsadek et al., 2014; Latzer et al., 2014; Meule et al., 2014; Tu et al., 2017), we used structural equation modeling (SEM), and statistical methods robust to nonnormality and categorical variables [a polychoric correlation matrix and a Mean-and Variance-adjusted Weighted Least Square (WLSMV) estimator for factor analysis, and ordinal alpha for measuring internal consistency of the questionnaire].

Furthermore, other aims of the present study were to assess: (i) the convergent validity of the I-NEQ-16 with questionnaires measuring binge eating, emotional and external eating, diurnal chronotype, insomnia, and anxiety and depression severity; and (ii) the discriminant ability of the I-NEQ-16 in determining the presence of NES.

While past research generally reported a multidimensional structure for the NEQ-14 (e.g., Moize et al., 2012; Elsadek et al., 2014; Latzer et al., 2014; Meule et al., 2014; Tu et al., 2017), we hypothesized that only a few items night be responsible for this heterogeneity and that, when excluding their effects, the structure of the I-NEQ-16 may be unidimensional. We also hypothesized good convergent validity with measures assessing sleep disturbances, emotional and eating psychopathology (e.g., positive associations with insomnia severity, depression and binge eating; and a satisfactory ability to discriminate between individuals with and without NES.

## Materials and Methods

### Participants and Procedure

The sample contained 482 Italian adults (380 women and 102 men) with a mean age of 25.5 years (*SD* = 10.9; range: 18–66 years). All participants were 18 years of age and older. We excluded participants with any condition affecting their ability to take the assessment (e.g., illiteracy) or denial of informed consent. The sample is described in Table 1.

**Table 1:** Characteristics of the sample (*n* = 482).

Variables	Percentages (Mean ± *SD*)
**SEX**	
Men	21.2%
Women	78.8%
Age – *M* (*SD*)	(25.48 **±** 10.88)
**MARITAL STATUS**	
Not-married (including widowed and divorced)	90.4%
Married	9.6%
**SCHOOL ATTAINMENT (NUMBER OF YEARS)**	
≤8 (elementary or junior high school degree)	0.6%
≤13 (high school degree)	70.3%
≥16 (Post-secondary degree)	29.1%
**JOB**	
Students	40.0%
Unemployed	2.7%
Other	57.3%
BMI – kg/m^2^	(22.01 **±** 3.43)
Underweight (BMI < 18.5)	8.1%
Normal weight (BMI = 18.5–24.9)	75.1%
Overweight (BMI = 25.0–29.9)	14.7%
Obese (BMI ≥ 30.0)	2.1%


*SD, Standard deviation, BMI, Body Mass Index.*

The sample was recruited through advertisements (i.e., flyers, newspaper, and online ads) for community groups and university campuses. After volunteering, individuals were approached by psychologists, informed about the aim of the study and informed on how to fill out the questionnaires using an online survey tool (Survio.com/it). Respondents received no payment and provided written, informed consent. The study protocol was approved by local research ethics review board (Ethics Committee of the European University of Rome, Rome, Italy).

### Measures

All participants were administered questions concerning their socio-demographic status (sex, age, marital status, job, and school attainment), and the Italian version of the NEQ-16, NEDQ, Binge Eating Scale (BES) (Gormally et al., 1982), Dutch Eating Behavior Questionnaire (DEBQ) (van Strien et al., 1986), Hospital Anxiety and Depression Scale (HADS) (Zigmond and Snaith, 1983; Iani et al., 2014), Athens Insomnia Scale (AIS) (Soldatos et al., 2000, 2003), and the Composite Scale of Morningness (CS) (Smith et al., 1989). Height and weight for calculating the BMI were self-rated.

The NEQ-16 is a 16-item questionnaire measuring NES severity. Items are rated on a 5-point Likert-type scale ranging between 0 and 4. Only items 1–9 are rated by all participants, while items 10–12 are answered by participants who wake up in the middle of the night for reasons other than using the bathroom, and items 13–16 are answered by participants who eat during nocturnal awakenings. All items except item 13 are summed to obtain a global score with higher scores indicating more severe NES. For the present study, two bilingual researchers adapted the Italian version of the questionnaire from the original English version using a back-translation procedure. The researchers agreed on the translation of all the items of the NEQ-16. Although an Italian version of the NEQ-14 has been recently proposed (Aloi et al., 2017), we began our study before the publication of this study. To create the I-NEQ-16 we used the well-established back-translation procedure.

The NEDQ is a 22-item self-report questionnaire used for the diagnosis of NES. Some items are dichotomous, others are rated on a Likert-type scale, while others are open-ended. The NEDQ assesses each of the 6 criteria for diagnosis of NES proposed by Allison et al. (2010), and it is used to place participants into four categories (no NES, mild NES, moderate NES, and full NES) according to how many symptoms are present. Considering that an Italian version of the NEDQ has not been adapted previously, two bilingual researchers adapted the Italian version of the questionnaire from the original English version using the same procedure used for the NEQ. The researchers agreed on the translation of all the items of the NEDQ.

The BES has 16-items assessing binge eating severity and the feelings and thoughts associated with the behavior and covert behavioral and cognitive/emotional manifestations of binge eating. Respondents have to choose between 3 or 4 response statements of increasing severity for each question. The BES successfully discriminated individuals with binge eating problems as judged by personal interviews (Gormally et al., 1982). BES scores range from 0 to 46, with higher scores indicating greater binge eating severity. The Italian version of the BES has been shown to have good psychometric properties (Di Bernardo et al., 1998; Imperatori et al., 2015). Cronbach alpha for the present sample was 0.86.

The Dutch Eating Behavior Questionnaire (DEBQ) is a 33-item questionnaire measuring three separate dimensions of eating behavior: emotional eating, external eating, and restrained eating. Items are rated on a five-point Likert-type scale (from 1, never to 5, very often). The Italian version of the DEBQ has been shown to have good psychometric properties (Dakanalis et al., 2013). In the present sample, we assessed only emotional eating (Cronbach alpha = 0.96) and external eating (Cronbach alpha = 0.87).

The HADS has 14-items and assesses symptoms of anxiety and depression. Items are rated on a 4-point Likert-type scale (from 0 to 3). Total scores range from 0 to 21 for each subscale, with greater scores reflecting greater anxiety and depression. The HADS was developed to screen for depression and anxiety in a hospital setting but has been used in the general population (Bjelland et al., 2002). The Cronbach alphas for the present sample were 0.83 and 0.72, respectively, for the anxiety and depression subscales.

The AIS is an 8-item questionnaire measuring insomnia severity. The first five items assess sleep induction, awakenings during the night, final awakening, total sleep duration, and sleep quality, and the final questions assesses sense of well-being, functioning (physical and mental), and sleepiness during the day (Soldatos et al., 2000). Items are rated on a 4-point Likert-type scale ranging between 0 and 3. Soldatos et al. (2000) and Soldatos et al. (2003) reported that the AIS has good psychometric properties. Cronbach alpha for the present sample was 0.82.

The CS is a 13-item questionnaire assessing individual differences in circadian rhythm and preferred hours for activity. Items of the CS are derived from the Morningness–Eveningness Questionnaire (Horne and Ostberg, 1976), and from the Diurnal Type Scale (Torsvall and Akerstedt, 1980), and are rated on a 4- or 5-point Likert-type scale. The Italian version of the CS has demonstrated good validity (Alzani and Natale, 1998; Natale and Alzani, 2001). Cronbach alpha for the present sample was 0.86.

### Statistical Analysis

In order to address the hypotheses of the present study, multiple statistical analyses were performed. Considering that Allison et al. (2008) considered item 13 only to be a screening tool for differentiating the NES from the parasomnia sleep-related eating disorder, and they did not considered it when calculating NEQ scores, we excluded this item from all the analyses.

In order to investigate/improve unidimensionality of the I-NEQ-16 (hypothesis 1) we calculated Ordinal alpha if an item is dropped. This statistic provides evidence of the possible presence of items increasing the heterogeneity of the measure. If ordinal alpha does not decrease after one item is dropped, this item should be considered problematic because it decreases content homogeneity of the questionnaire and should be removed if unidimensionality is a goal. Ordinal alpha coefficients (α) were also reported as measures of reliability (Zumbo et al., 2007). Ordinal alpha may estimate reliability more accurately than Cronbach alpha for questionnaires using Likert-type items. In fact, the calculation of Cronbach alpha uses the Pearson covariance matrix whose assumption is that the data are continuous. When the assumption is violated, the Pearson covariance matrix severely underestimates the true relationship between the variables. In this case, the use of a polychoric correlation matrix and ordinal alpha more accurately estimate the relationship between the underlying variables (Carroll, 1961; Gadermann et al., 2012).

After removing problematic items, the unidimensionality of the I-NEQ-16 was tested using confirmatory factor analysis with a WLSMV estimator. Model fit was assessed using the following indices: (1) the Root Mean Square Error of Approximation (RMSEA), with values between 0.05 and 0.08 indicative of adequacy of the model, and values below 0.05 indicating evidence of good fit (Browne and Cudek, 1993; Hu and Bentler, 1999); (2) the Comparative Fit Index (CFI), with values greater than 0.95 indicating good fit of the model; (3) the Weighted Root Mean Square Residual (WRMR), with values of less than 1.0 indicating good fit (Yu, 2002); and (4) a nonsignificant χ^2^-test (*p* > 0.05), although the χ^2^-test over-rejects true models for large samples. Modification indices were used to evaluate localized areas of strain in the solution and to suggest improvements to the final model.

In order to investigate the convergent validity of the I-NEQ-16 (i.e., hypothesis 2), Spearman *rho* correlation coefficients were reported as measures of associations between NEQ scores and BMI, binge eating (BES), emotional and external eating (DEBQ), diurnal chronotype (CS), insomnia (AIS), and anxiety and depression severity (HADS). Spearman *rho* coefficients were preferred over Pearson’s *r* because some variables deviated significantly from normality (skewness or kurtosis > 1).

Finally, in order to assess the performance of the I-NEQ-16 in categorizing individuals based on the severity of their current NES as diagnosed according the NEDQ (i.e., hypothesis 3), we performed a series of ROC (Receiver Operating Characteristics) test procedures. Furthermore, following Runfola et al. (2014), we also calculated partial correlations between NEQ scores and concurrent measures while controlling for the effect of binge eating severity. It is important to control for the effect of binge eating severity when measuring the association between the I-NEQ-16 and other variables because night eating and binge eating are highly comorbid and the latter could partially or totally explain the association that night eating severity has with emotional and eating psychopathology.

All the analyses were performed with the Statistical Package for the Social Sciences (SPSS) 19.0 for Windows, and Mplus 7.0 (Muthén and Muthén, 1998–2010). R version 3.4.2 (The R foundation for Statistical Computing, 2017) was used for item analysis and for calculating ordinal alpha.

## Results

### Item Analysis

Ordinal alpha for the Italian version of the NEQ-16 was 0.85 but, after items 1 and 7 were dropped, alpha increased to 0.87 and 0.86, respectively. Furthermore, when item 4 was dropped, ordinal alpha for the remaining items did not decrease. Considering that these items are a source of heterogeneity and that the dimension rated with these items is also rated with other items, we performed further analyses without them. In addition, item 13 was not included in the analyses because it is considered to be only a screening tool for differentiating the NES from the parasomnia sleep-related eating disorder and it is not included in the global score. Using SEM, we tested the fit of a one-factor model for the remaining 12 items of the I-NEQ-16.

Confirmatory factor analysis testing the unidimensionality of the I-NEQ-16 indicated that this model does not completely fit the data (χ^2^_54_ = 226.75, *p* < 0.001; RMSEA = 0.082, 90% CI = 0.071/0.093; CFI = 0.959; WRMR = 1.494), while modification indices indicated the presence of localized areas of strain and suggested allowing correlated errors between six pairs of items. Considering that the use of modification indices is a data-driven approach and, the more changes that are made to the model, the more the possibility of replicating the solution in another sample is decreased (Landis et al., 2009), we allowed correlated errors only for the first three pairs of items (between items 3 and 5, both assessing hyperphagia after suppertime, items 6 and 8 assessing depressed mood and insomnia, and items 15 and 16 both assessing distress due to night eating. We did not allow correlated errors between items 8 “trouble getting to sleep” and 9 “get up in the middle of the night,” 8 and 16 “how much night eating is affecting life,” or 3 “cravings after supper” and 16), until all fit indices (except for the χ^2^ test as expected) were suggestive of a good fit for the model (χ^2^_51_ = 113.83, *p* < 0.001; RMSEA = 0.051, 90% CI = 0.038/0.064; CFI = 0.985; WRMR = 0.967) (factor loadings are reported in Table 2).

**Table 2:** Standardized factor loadings for the I-NEQ-16 (*n* = 482).

Items	λ	Items	λ
NEQ2 -	0.455	NEQ10 –	0.924
“When do you usually eat for the first time?”		“Do you have cravings or urges to eat snacks when you wake up at night?”	
“Quando mangi per la prima volta di solito?”		“Hai una forte voglia di mangiare degli snack quando ti svegli la notte?”	
NEQ3 -	0.440	NEQ11 –	0.956
“Do you have cravings or urges to eat snacks after supper, but before bedtime?”		“Do you need to eat in order to get back to sleep when you awake at night?”	
“Hai una forte voglia di mangiare degli snack dopo cena ma prima dell’ora di andare a letto?”		“Quando ti svegli la notte, hai bisogno di mangiare per tornare a dormire?”	
NEQ5 -	0.452	NEQ12 –	0.967
“How much of your daily food intake do you consume after suppertime?”		“When you get up in the middle of the night, how often do you snack?”	
“Quanto del cibo che mangi quotidianamente lo consumi dopo cena?”		“Quando ti alzi nel bel mezzo della notte, quanto spesso fai uno spuntino?”	
NEQ6 -	0.362	NEQ14 –	0.962
“Are you currently feeling blue or down in the dumps?”		“How much control do you have over your eating while you are up at night?”	
“Ti senti depresso o molto triste al momento?”		“Quanto riesci a controllare il tuo mangiare mentre sei in piedi durante la notte?”	
NEQ8 –	0.481	NEQ15 –	0.938
“How often do you have trouble getting to sleep?” “Quanto spesso hai difficoltà ad addormentarti?”		“How upsetting is your night eating to you?” “Quanto ti senti turbato dal tuo mangiare di notte?”	
NEQ9 –	0.661	NEQ16 –	0.782
“Other than only to use the bathroom, how often do you get up at least once in the middle of the night?”		“How much has your night eating affected your life?”	
“Escludendo le volte in cui ti alzi solo per andare in bagno, quanto spesso ti alzi nel cuore della notte?”		“Quanto ha inciso sulla tua vita il fatto che mangi di notte?”	
Ordinal alpha		0.89	
*λ, factor loadings.*			


### Psychometric Properties of the I-NEQ-16

The ordinal alpha for the I-NEQ-16 was 0.89. The average score was 5.07 (*SD* = 4.36; range = 0/27), with no sex differences (*t*_480_ = -1.37, *p* = 0.17) (see Table 3 for descriptive statistics for all the measures broken down by sex), and a significant but weak association with age (*r* = -0.23, *p* < 0.001).

**Table 3:** Descriptive statistics for all the measures administered broken down by sex (*n* = 482).

Variables	Men *N* = 102	Women *N* = 380	*t* (degree of freedom = 480)	*P* <
I-NEQ-16 – *M* ± *SD*	4.55 ± 5.09	5.21 ± 4.14	-1.37	0.17
BES – *M* ± *SD*	5.85 ± 5.95	8.13 ± 6.81	-3.07	0.01
Emotional_eating – *M* ± *SD*	24.03 ± 10.96	29.14 ± 11.21	-4.11	<0.001
External_eating – *M* ± *SD*	27.57 ± 6.76	29.69 ± 6.54	-2.89	0.01
CS – *M* ± *SD*	33.83 ± 6.78	33.25 ± 7.04	0.75	0.45
AIS – *M* ± *SD*	4.64 ± 3.73	5.32 ± 3.86	-1.59	0.11
HADS-D – *M* ± *SD*	3.90 ± 3.06	5.14 ± 3.53	-3.24	0.001
HADS-A – *M* ± *SD*	5.57 ± 3.74	7.51 ± 4.09	-4.34	<0.001


*BES, Binge Eating Scale; CS, Composite Scale of Morningness; AIS, Athens Insomnia Scale; HADS-D, Hospital Anxiety and Depression Scale, Depression; HADS-A, Hospital Anxiety and Depression Scale, Anxiety; BMI, Body Mass Index. I-NEQ-16, Italian version of the Nignt Eating Questionnaire-16.*

Detailed correlations between variables are reported in Table 4. The I-NEQ-16 global score was significantly associated with concurrent measures: BES (*rho* = 0.42, *p* < 0.01), DEBQ emotional and external eating (respectively, *rho* = 0.23, *p* < 0.01 and *rho* = 0.35, *p* < 0.01), HADS depression (*rho* = 0.43, *p* < 0.01) and anxiety (*rho* = 0.52, *p* < 0.01), CS (*rho* = -0.39, *p* < 0.01), and AIS (*rho* = 0.60, *p* < 0.01) scores.

**Table 4:** Correlations between measures (*n* = 482).

	BES	Emotional eating	External eating	CS	AIS	HADS-D	HADS-A	BMI
I-NEQ-16	0.418^∗∗^	0.234^∗∗^	0.353^∗∗^	-0.392^∗∗^	0.600^∗∗^	0.428^∗∗^	0.516^∗∗^	0.013
BES	–	0.558^∗∗^	0.394^∗∗^	-0.171^∗∗^	0.417^∗∗^	0.467^∗∗^	0.451^∗∗^	0.187^∗∗^


*^∗∗^ Significant for p < 0.01; ^∗^Significant for p < 0.05. BES, Binge Eating Scale; CS, Composite Scale of Morningness; AIS, Athens Insomnia Scale; HADS-D, Hospital Anxiety and Depression Scale, Depression; HADS-A, Hospital Anxiety and Depression Scale, Anxiety; BMI, Body Mass Index. I-NEQ-16, Italian version of the Nignt Eating Questionnaire-16.*

However, when controlling for the effect of binge eating severity, the correlation with emotional eating was not significant (*rho* = -0.007, *p* = 0.88), and the effect size of the other correlations was reduced (the highest correlation was with insomnia severity complaints, *rho* = 0.53, *p* < 0.01). The BMI was not associated significantly with I-NEQ-16 scores (*rho* = -0.01; *p* = 0.78) before controlling for BES scores, and it was weakly but significantly associated with I-NEQ-16 scores after controlling for the effect of binge eating severity (*rho* = -0.11, *p* < 0.05).

On the NEDQ, four individuals received the diagnosis of NES ( = 0.8%). I-NEQ-16 scores were able to categorize individuals according to their diagnosis of NES on the NEDQ [area under the curve (AUC) = 0.984, 95%CI = 0.958/1.000, *p* = 0.001]. A score of 12 and higher on the I-NEQ-16 had a sensitivity of 100% and a specificity of 94% in detecting people with NES. Thirty-five individuals (7.3%) had scores of 12 and higher on the I-NEQ-16, including all the individuals who received a diagnosis of NES on the NEDQ. Comparing these results with those of the original NEQ-14 version (AUC = 0.947, 95%CI = 0.880/1.000, *p* = 0.001; sensitivity 50% and specificity 98.5% for the cutoff score of 25), the I-NEQ-16 seems to categorize individuals according to their diagnosis of NES on the NEDQ slightly better than the does the NEQ-14. Nevertheless, these results are based only on four individuals with an NES diagnosis, and different results could arise from larger samples with more people at risk of NES.

## Discussion

The main aim of the present study was to create a unidimensional Italian version of the NEQ-16 (I-NEQ-16) which included the items proposed by Allison et al. (2008) for assessing the distress associated with night eating. Item analysis indicated that items 1, 4, and 7 were lowering the internal consistency of the questionnaire, and so they were removed in order to reach unidimensionality. These results are consistent with those reported by Aloi et al. (2017) that investigated an Italian version of the NEQ-14 and reported low item-total correlation and factor loading for the item 9. Using a confirmatory/exploratory approach (i.e., a confirmatory factor analysis with the use of modification indices), we found that a modified one-factor model could represent the latent structure of the I-NEQ-16 quite well, after including correlations between three pairs of items which measured similar dimensions of night eating. Supporting our solution was the fact that we found a higher internal consistency for the I-NEQ-16 (ordinal alpha = 0.89) than those reported by Allison et al. (2008) for the NEQ-14, and from Aloi et al. (2017) for the Italian version of the NEQ-14.

In the past, researchers (Allison et al., 2008; Moize et al., 2012; Elsadek et al., 2014; Latzer et al., 2014; Meule et al., 2014; Aloi et al., 2017; Tu et al., 2017) have investigated only the factor structure and psychometric properties of the NEQ-14, and they reported either a hierarchical four-factor structure or a multidimensional structure with 4 or 5 first-order factors (Moize et al., 2012; Elsadek et al., 2014; Latzer et al., 2014; Meule et al., 2014; Tu et al., 2017). Nevertheless, they all suggested the use of a global score to rate NES severity which is based on the hypothesis of unidimensionality of the scale. Therefore, our study is the first one investigating the factor structure, the unidimensionality, and the psychometric properties of the NEQ-16.

Consistent with the results of previous studies which administered the NEQ-14 (Vander Wal, 2012; Aloi et al., 2017), I-NEQ-16 scores were significantly associated with concurrent scores for binge eating, emotional and external eating, depressive and anxiety symptoms, and sleep-related problems (i.e., nocturnal chronotype and insomnia severity). Considering that NES is frequently comorbid with binge eating (Napolitano et al., 2001; Grilo and Masheb, 2004; Colles et al., 2007; Allison et al., 2008; McCuen-Wurst et al., 2018) and that binge eating severity has been associated with emotional psychopathology (Bulik et al., 2002) and insomnia (Kenny et al., 2018), we also calculated partial correlations between the I-NEQ-16 and concurrent measures, while controlling for the effect of binge eating severity, and found that this variable was not able to account for all the associations night eating severity has with anxiety, depression, and sleep-related problems (Runfola et al., 2014). Furthermore, the BMI score was weakly but statistically significantly associated with I-NEQ-16 scores only after controlling for the effect of binge eating severity. Runfola et al. (2014), in a sample of university students, reported that those with NES and those without night eating did not differ in BMI, despite the fact that students with NES were more likely to have histories of underweight and anorexia nervosa.

I-NEQ-16 scores were able to categorize individuals according to their diagnosis of NES (using the NEDQ) slightly better than the NEQ-14, although the low number of positives on the NEDQ prevent us from generalizing this result to other samples. In our sample only 0.8% of the respondents had a diagnosis of NES according to the NEDQ while 7.3% were screened as positive on the I-NEQ-16 using a cutoff score of 12 and higher on the I-NEQ-16. A similar comparison was reported from Nolan and Geliebter (2017), although they reported data only for individuals who had at the same time a score of 25 and higher on the NEQ-14 and reported distress associated with night eating on the NEDQ. In their study, 4.6% of participants scored ≥25 on the NEQ-14 and expressed distress or impairment associated with their night eating (5 of them did not meet the criteria on the NEDQ), while 6.9% of participants met the criteria for NES on the NEDQ (22 of them did not reach the threshold on the NEQ-14). Probably, if the authors had used only NEQ-14 scores to screen for NES, more people not meeting criteria on the NEDQ would have been labeled as positive on the NEQ-14 as in our study. Nevertheless, if levels of sensitivity/specificity found in our study are replicated in future studies, the I-NEQ-16 could be considered to be a good measure for the screening of NES in the general population.

Our results must be considered in light of some issues referred to the design of the study. First, in our sample there was a high proportion of female participants, probably associated with the recruitment of university students. Second, our results are based on a sample of mostly young adults which limits the generalizability of these findings to clinical samples and older adults. Third, although the sample could be considered quite large, few people met the criteria for NES, and the results based on the ROC curves should be considered only a first step in the process of cross-cultural validation of the I-NEQ-16. Fourth, we administered only self-report measures which are potentially affected by social desirability. Fifth, we administered a nonvalidated version of the NEDQ to assess independently from the I-NEQ-16 the presence of NES in our sample. Unfortunately, in Italy there are not validated measures for the diagnosis of the NES. Nevertheless, our study adds to the results of previous studies which investigated the dimensionality and psychometric properties of the NEQ-14. We used golden-standard statistical procedures (i.e., SEM with correlation matrix and an estimator robust for categorical items) and directly tested the unidimensionality of a version of the NEQ which included additional items rating the distress associated with night eating (NEQ-16). Nevertheless, the use of ordinal alpha to exclude items from the I-NEQ-16 is a data-driven approach and at least one verification sample independent from the one used in the present study is necessary to confirm the factor solution found in our study.

## Conclusion

Our results indicate that the I-NEQ-16 is a unidimensional measure, thereby supporting the use of a global score for rating the severity of NES in the general population. The I-NEQ-16 has good internal consistency and discriminant validity. Nevertheless, factor structure should be confirmed in future studies with independent samples, and an appropriate threshold value for screening for the presence of NES should be investigated with larger samples. It may be concluded that the I-NEQ-16 is potentially a valid measure useful for investigating correlates of night eating in the general population.

## Ethics Statement

All procedures performed in studies involving human participants were in accordance with the ethical standards of the institutional and/or national research committee and with the 1964 Helsinki declaration and its later amendments or comparable ethical standards.

## Author Contributions

MI was involved in the study conception and design, and analysis and interpretation of data. LG and MB were involved in the study conception and design, and acquisition of data. All the authors were involved in the interpretation of data and the critical revision of the manuscript, and they all provided final approval of the version to be published.

## Conflict of Interest Statement

The authors declare that the research was conducted in the absence of any commercial or financial relationships that could be construed as a potential conflict of interest.
